# Photoionization, Structures, and Energetics of Na‐Doped Formic Acid–Water Clusters

**DOI:** 10.1002/cphc.202100861

**Published:** 2022-01-27

**Authors:** Attila Bende, Maria F. Gaele, Tonia M. Di Palma

**Affiliations:** ^1^ Istituto di Scienze e Tecnologie per l'Energia e la Mobilità Sostenibili (STEMS)-National Research Council of Italy Via Marconi 4 80125 Napoli Italy; ^2^ Molecular and Biomolecular Physics Department National Institute for R&D of Isotopic and Molecular Technologies Donat Street, No 67–103 RO-400293 Cluj-Napoca Romania

**Keywords:** formic acid, sodium-doped clusters, Rydberg electronic states, intermolecular energy decomposition, mass and PIE spectra

## Abstract

The influence of formic acid on water cluster aggregation has been investigated experimentally by mass spectrometry and tunable UV laser ionization applied to Na‐doped clusters formed in the supersonic expansion of water vapors seeded with formic acid (FA) as well as theoretically using high level quantum chemistry methods. The mass spectra of Na−FA(H_2_O)_n_ clusters show an enlarging of mass distribution toward heavier clusters with respect to the Na−(H_2_O)_n_ clusters, suggesting similar mass distribution in neutral clusters and an influence of formic acid in water aggregation. Density functional theory and coupled‐cluster type (DLPNO‐CCSD(T)) calculations have been used to calculate structures and energetics of neutral and ionized Na−FA(H_2_O)_n_ as well as neutral FA(H_2_O)_n_. Na‐doped clusters are characterized by very stable geometries. The theoretical adiabatic ionization potential values match pretty well the measured appearance energies and the calculated first six electronic excited states show Rydberg‐type characters, indicating possible autoionization contributions in the mass spectra. Finally, theoretical calculations on neutral FA(H_2_O)_n_ clusters show the possibility of similarly stable structures in small clusters containing up to n=4–5 water molecules, where FA interacts significantly with waters. This suggests that FA can compete with water molecules in the starting stage of the aggregation process, by forming stable nucleation seed.

## Graphical abstract

**Cluster formation**: Water aggregation in presence of formic acid at low concentration was investigated by mass spectrometry/photoionization spectroscopy. Experimental data show the formation of larger clusters in the presence of acid molecules. DFT and coupled‐cluster type (DLPNO‐CCSD(T)) calculations support experimental results and suggest that water molecules aggregate preferentially with acid molecule but mainly in the first stage of cluster formation.

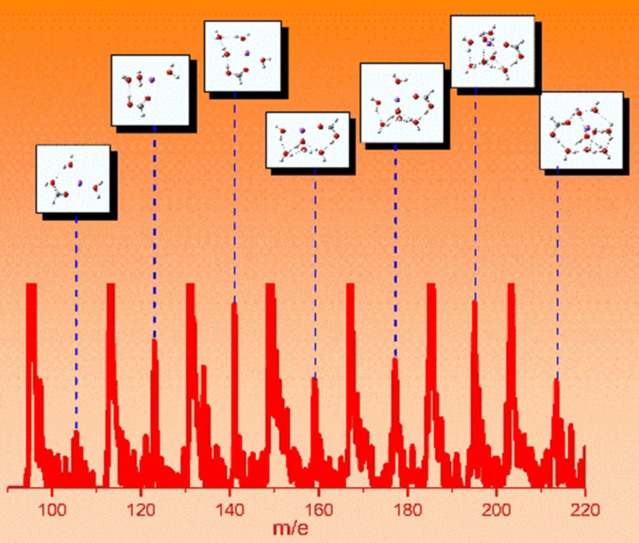

## Introduction

Many reactions occurring in the atmosphere involving nucleation and aerosol growth are poorly understood due to the lacking of experimental method able to study directly the process. The key understanding of nucleation phenomena are the structures and properties of the smallest molecular aggregates formed in the initial stage of the process. Studies on structure and dynamics of clusters carried out in molecular beam experiments are particularly suitable for this purpose. In particular water clusters have been used to successfully model the reactions that occur on cloud and ice surfaces.^[1]^ In the same way, water clusters mixed with a foreign molecule constitute a model to study the initial phase of heteromolecular nucleation in the atmosphere^[2]^ and then the aerosol formation affected by anthropogenic and biogenic emissions. Formic acid (FA), the simplest carboxylic acid, is ubiquitous in the atmosphere and is introduced from a variety of sources, from vegetation to burned biomass and urban pollution.^[3]^ As other organic acids, FA contributes to the formation of cloud condensation nuclei.^[4]^ Moreover, it is reported FA stabilizes clusters of water with important nucleation precursors^[5]^ and that its presence in small pure water clusters significantly affects the hydrogen bond between water molecules, making water in the cluster able to bond more strongly adjacent water molecules. Then, was postulated hydrated FA clusters could grow more easily than pure water clusters.^[6]^ FA clusters are mainly studied using mass spectrometric methods detecting protonated species mainly resulting from fragmentation processes.[^7^, ^8^, ^9^] Information on neutral acid‐water cluster mass distribution may be obtained, in some cases, with alkali metal doping methods and UV photoionization. The concept of the method relies on the fact that the ionization potential of a complex with an alkali metal atom stuck on the cluster is significantly lowered and close to that of metal atom. Therefore, Na‐doped clusters can be easily detected by using commercial UV laser as photoionization sources in mass spectrometers. There are clusters, which cannot be detected by the Na‐doping method, e. g. strong acid doped clusters, as nitric acid, where an ion pair Na^+^−HNO_3_^−^ is formed in the cluster. As a result, the clusters have high ionization energy so they are not detected in the UV photoionization experiments.^[10]^

Although in cluster experiments the fragmentation can never be completely ruled out, the method Na‐doping/UV photoionization has been proved to be almost fragmentation‐free,^[11]^ at least for some hydrogen bonded clusters such as water clusters or carboxylic acid clusters.^[12]^ We have recently demonstrated that acetic acid seeded in water vapor and expanded in supersonic jet, under specific expansion conditions, enhances cluster growth.^[13]^ In addition, theoretical calculations showed that small acid–water clusters are stable and their formation is thermodynamically favored with respect to pure water clusters, especially at lower temperatures, suggesting that acetic acid could increase aerosol formation in moist air by easily forming pre‐nucleation embryos.^[13]^

Here we present a study on Formic acid‐water clusters by using the experimental method of Na‐doping combined with mass spectrometry. We adopted the experimental method of Na‐doping and tunable UV ionization to get information on classes of non‐fragmented neutral clusters and on their ionization energy. DFT and coupled cluster calculations have been applied to study structures and energetics of neutral and ionized Na−FA(H_2_O)_n_ as well as neutral FA(H_2_O)_n_ clusters. Absorption spectra have been also calculated in order to elucidate the nature of the early stage of the ionization process of sodium doped clusters.

## Results and Discussion

In the supersonic expansion in the vacuum, carboxylic acid vapors easily aggregate forming pure acid or mixed acid‐water clusters detected in our apparatus as Na‐doped clusters by laser UV ionization.[^13^, ^14^, ^15^] In particular, in ref [14] we demonstrated that the ion signals of Na−(FA)_n_ are produced by one‐photon ionization, so the cluster ionization potentials were correctly assigned in the laser UV range. In the same paper, we showed that the photoionization efficiency (PIE) spectra of smaller clusters did not change by recording signals at different delay supersonic valve opening – laser firing, from the beam onset to higher delay. In these conditions, we sampled different cluster size distributions and so fragmentation effects, if any, from larger clusters on PIE signals of smaller clusters. We obtained the same PIE spectra in different beam sampling conditions, indicating negligible contributions from larger cluster fragmentation and so demonstrating that Na‐doping for these clusters is an effective “soft“ ionization method.

However, since in cluster experiments fragmentation contributions can never be completely excluded, to simplify the mass spectra and to avoid contributions of fragmented higher masses signals on the appearance energy measurements of clusters of interest, the measurements were performed by considering the vapor clustering of aqueous solution with low mole fraction of FA. In fact, it is expected that the acid percentage in the vapors of solutions with low acid concentrations is equally low. In particular, the percentage of FA in the vapors of liquid solutions with 0.02 M of acid is expected approximately ≈2 %.^[16]^ As results, the mass spectra are simplified, pure (FA)_n_ or mixed (FA)_m_(H_2_O)_n_ clusters are not formed, but the Na−FA(H_2_O)_n_ are detected as low intensity peaks. Nevertheless, it was still possible to evaluate the appearance energy of these clusters although with a signal/noise ratio lower than that of Na−(FA)_n_ or Na−(H_2_O)_n_ clusters.[^14^, ^17^]

**Figure 1:**
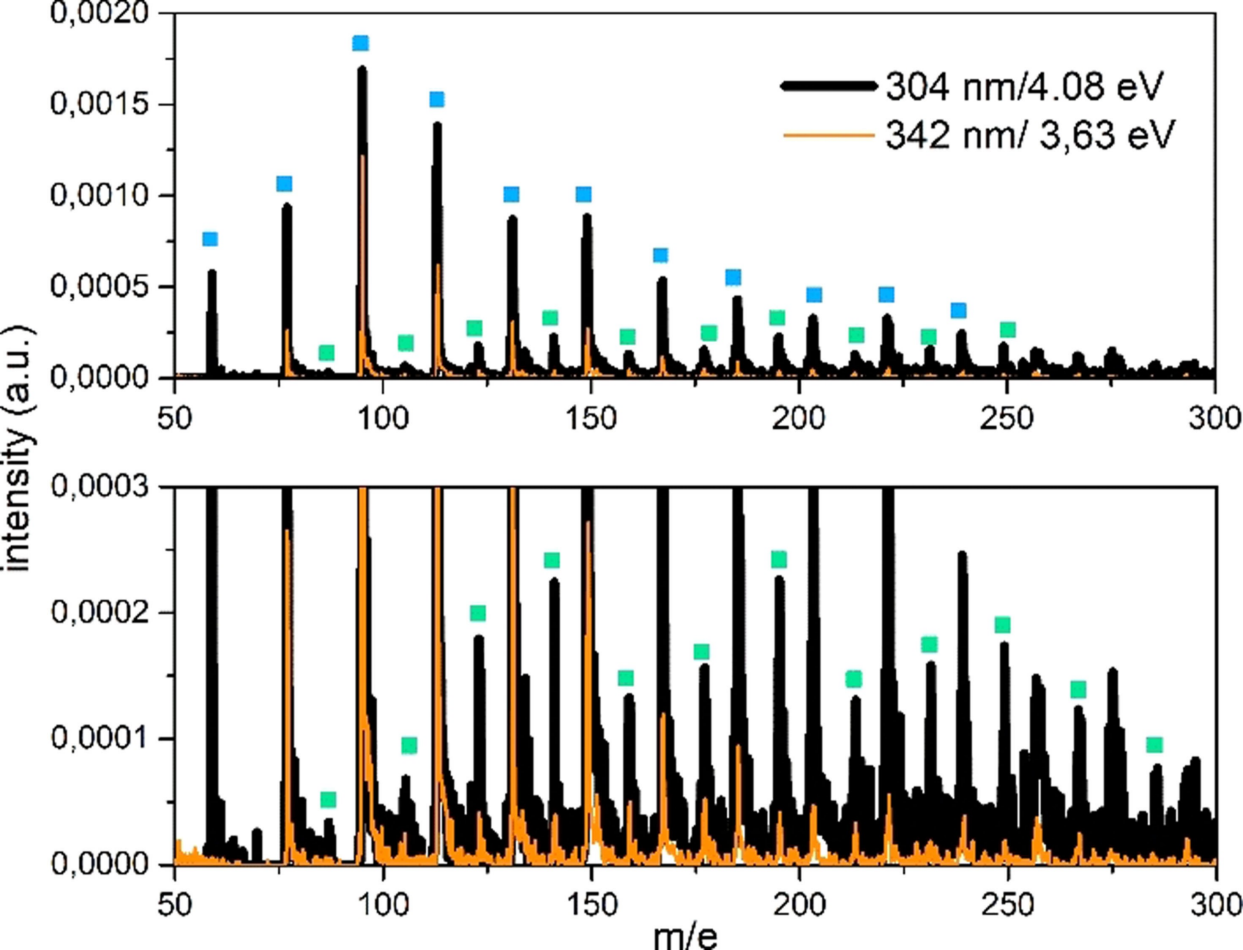
Mass spectra of doped cluster distribution at 4.08 and 3.63 eV. Blue squares indicate Na−(H_2_O)_n_ clusters. Green squares indicate Na−FA(H_2_O)_n_ clusters. The bottom panel is an enlarged view of the upper one. The spectra are reported in the same a.u. y‐scale. Expanded spectra are reported in Figures S5a and S5b.

In Figure 1 the mass spectra acquired above (dark line) and at the threshold (orange line) ionization energy of most Na−FA(H_2_O)_n_ clusters are reported. The Na−FA(H_2_O)_n_ cluster peaks are marked with green squares. The spectra have been acquired 20 μs after the molecular beam onset. The Na−FA(H_2_O)_n_ mass distribution does not show the same mass distribution of doped water clusters. Instead, also at ionization threshold, it shows increased peak intensity towards larger masses. Since our experimental conditions have been set to reduce fragmentation effects on the intensity of the detected ion signals, differences in the mass distribution can be attribute to the presence of the acid molecule which influences the physicochemical properties of the clusters and their growth.

**Figure 2:**
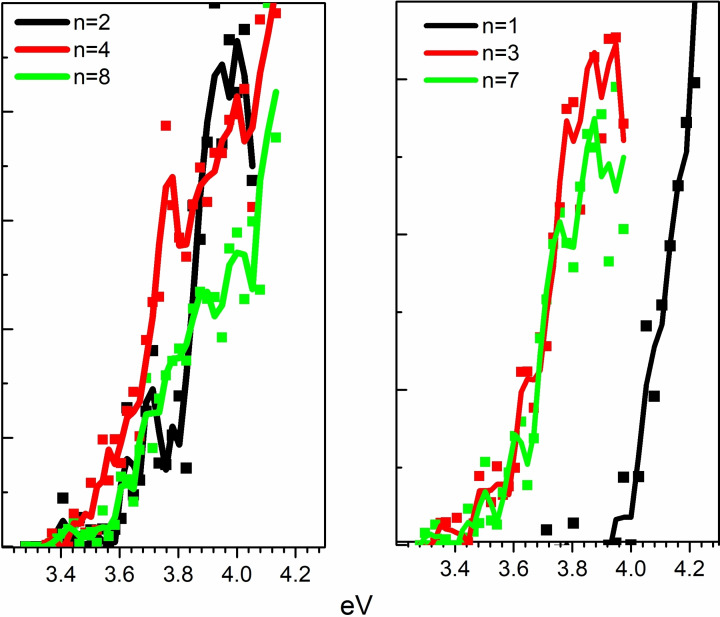
PIE spectra of some Na−FA(H_2_O)_n_ clusters. Each spectrum is normalized to its maximum. The spectra are reported in the same a.u. y‐scale. The continuous lines are smoothed profiles of each spectrum.

The photoionization efficiency spectra of different Na−FA(H_2_O)_n_ clusters are reported in Figure 2. Each spectrum has been normalized to its maximum and the appearance energies have been retrieved from the wavelength corresponding to the 10 % of the maximum. The PIE spectra of smaller clusters (n≤2) have been acquired on the beam front as their signals are more intense.^[14]^ From the figure it is clear a shift of the spectrum onset from n=1 to n=2. From n≥2 the spectra onsets are between 3.5 and 3.6 eV. We have already found appearance energies in the UV range for similar classes of clusters. These appearance energies have been attributed to clusters characterized by adiabatic ionization energies in the same UV energy range of the PIE spectra.^[14]^ In fact, the adiabatic photoionization implies a significant rearrangement of the ionic core and formation of electronic Rydberg‐type excited states that could give rise to autoionization during orbital relaxing. Therefore, clusters with calculated adiabatic ionization energy close to the observed appearance energy have to be also considered in the data interpretation. In Table 3 the appearance energy values of clusters Na−FA(H_2_O)_n_ with n up to 8 are reported together with the calculated adiabatic ionization energy values as well as the calculated adiabatic ionization energy including thermal effects as found for the corresponding minimum energy cluster structures (see next paragraph). As it clearly results from the figure, the appearance energies match very well the calculated adiabatic ionization energies, suggesting contributions in the ion signals from autoionization phenomena.

### Cluster Structures and Ionization Potentials

Ab initio calculations at the MN15/def2‐TZVPD level of theory were performed for the neutral and ionic sodium doped clusters containing, in addition, a single formic acid and up to 8 water molecules. For both neutral and ionic species, several starting geometry configurations were generated in order to identify the global and different local equilibrium geometries. For the global and its close‐lying local geometry cases the enthalpy (zero‐point vibrational energy+thermal energy correction for translation, vibration, and rotation) and entropy corrections based on the harmonic vibrational analysis were also computed. The VIE (or Vertical Ionization Energy, given in eV unit) was computed at the same MN15/def2‐TZVPD level of theory and is considered as the difference between the total electronic energies of the neutral and ionic electron configurations both of them taken in the equilibrium geometry of the neutral species. The energy‐based AIE (or Adiabatic Ionization Energy, given in eV unit) was calculated as the difference between the total electronic energies of the neutral and ionic electron configurations each of them taken in their equilibrium geometry configuration. The enthalpy‐based AIE (henceforth denoted as AIE* and given in eV unit) was calculated as the difference between the lowest enthalpies obtained for the neutral and ionic geometry configurations. Similarly, the Gibbs free energy‐based AIE (henceforth denoted as AIE** and given in eV unit) was calculated as the difference between the lowest Gibbs free energies (both the enthalpy and entropy effects) obtained for the neutral and ionic geometry configurations. The intermolecular interaction energy (denoted as ΔE and given in kcal/mol) is defined based on the cluster dissociation concept and is computed as the difference between the total electronic energy at supramolecular level and the sum of isolated molecular electronic energies obtained for each cluster component. These types of intermolecular interaction energies are denoted with the “Δ” prefix. In turn, to estimate the strength of the cluster cohesion grown around the sodium atom, the total intermolecular interaction energy can be decomposed into the pair contributions between different cluster constituents, using the LED (or Local Energy Decomposition) method defined in the framework of the DLPNO‐CCSD(T) /def2‐TZVPD theory (for details, see Computational Details section). Based on the localization of the occupied pair natural orbitals the total energy (reference energy+correlation) can be decomposed into intra‐ and inter‐fragment contributions as [Eq. 1]:

(1) $$DE={\delta E}_{el-prep}^{ref.}+{DE}_{elstat.}^{ref.}+{DE}_{exch.}^{ref.}+{DE}_{non-disp.}^{C-CCSD}+{DE}_{disp.}^{C-CCSD}+{DE}_{int}^{C-\left(T\right)}$$

where, ${\delta E}_{el-prep}^{ref.}$ means the electronic preparation (or intra‐fragment reference) energy and describes how much energy is necessary to bring the fragments into the electronic structure that is optimal for interaction, ${DE}_{elstat.}^{ref.}$ and ${DE}_{exch.}^{ref.}$ are the inter‐fragment electrostatic and exchange contributions, ${DE}_{non-disp.}^{C-CCSD}$ and ${DE}_{disp.}^{C-CCSD}$ are the non‐dispersive and dispersive parts of the correlation energy at CCSD (coupled‐cluster with single and double excitation), as well as ${DE}_{int}^{C-\left(T\right)}$ is the triples correction term to the inter‐fragment interaction energy. It is easy to see that, while the first component can be associated to each cluster fragment, the others are related to pair interactions between different fragments. Since, we are not explicitly interested in the nature of the interaction, but in how strong they are between the different cluster components, the energy components were grouped based on their intra‐ or inter‐fragment character and the contribution of the intra‐fragments is given as a single energy sum, called ${DE}_{intra}$ . Accordingly, it can be easily estimated the intermolecular interaction energies (denoted as DE and given in kcal/mol) between sodium – formic acid, sodium‐waters, formic acid – waters and water – water both for neutral and ionic cluster species. One needs to mention, that not only the electronic deformation energy is important but also the so‐called geometry deformation energy (or ${\Delta E}_{def.}^{geom}$ ) which is the energy needed to distort the fragments from their equilibrium configuration to the interacting geometry. The Na⋅⋅⋅O interatomic and C=O bond distances for neutral and ionic forms as well as the VIE, AIE, AIE* and AIE** ionization energies computed for Na−FA(H_2_O)_n_ (n=0–8) mixed clusters at MN15/def2‐TZVPD level of theory are collected in Table 1, while the interaction energies between different cluster components as well as their geometry distortion and intra‐fragment contributions for both neutral and ionic cases computed at DLPNO‐CCSD(T)/def2‐TZVPD theory based on the LED decomposition scheme are given in Table 2. Geometry figures of different cluster configurations are presented in Tables 3 and 4 as well as in Figure S1 of the Supporting Information. The total intermolecular interaction energies (ΔE), enthalpies (ΔH) and Gibbs free energies (ΔG) of the Na⋅⋅⋅FA⋅⋅⋅(H_2_O)_n_ (n=0–8) mixed clusters obtained at MN15/def2‐TZVPD level of theory considering T=298.15 K are given in Table S1 in the Supporting Information (SI).

**Table 1:** The Na⋅⋅⋅O interatomic distance of the Na−FA contact and the C=O bond length (in Å) for neutral and ionic forms as well as the VIE, AIE, AIE* and AIE** ionization energies (in eV) computed for Na⋅⋅⋅FA⋅⋅⋅(H_2_O)_n_ (n=0–8) mixed clusters at MN15/def2‐TZVPD level of theory. AE(exp) are the experimental appearance energies (in eV).

Nr. H_2_O	Species	d(Na⋅⋅⋅O)	d(C=O)	VIE	AIE	AIE*	AIE**	AE(exp)
0	neut.	2.397	1.208	4.37	4.01	4.03	4.08	4.3
	ion.	2.179	1.219					
1	neut.	2.332	1.213	4.12	3.78	3.94	3.93	4.05
	ion.	2.199	1.216					
2	neut.	2.405	1.297	5.73	3.64	3.74	3.60	3.6
	ion.	2.294	1.203					
3	neut.	2.329	1.296	5.88	3.59	3.68	3.61	3.59
	ion.	2.293	1.217					
4	neut.	2.432	1.297	5.99	3.57	3.66	3.51	3.52
	ion.	2.277	1.213					
5	neut.	2.437	1.301	6.05	3.47	3.57	3.48	3.56
	ion.	2.233	1.217					
6	neut.	2.381	1.318	5.95	3.49	3.57	3.44	3.58
	ion.	2.347	1.217					
7	neut.	2.404	1.319	6.04	3.46	3.55	3.49	3.58
	ion.	2.361	1.220					
8	neut.	2.423	1.320	5.98	3.44	3.52	3.50	3.6
	ion.	2.426	1.223					

**Table 2:** The total interaction energies (ΔE, in kcal/mol) and interaction energy components between different cluster constituents (DE, in kcal/mol) for both neutral and ionic forms of the Na⋅⋅⋅FA⋅⋅⋅(H_2_O)_n_ (*n*=0–8) mixed clusters obtained at DLPNO‐CCSD(T)/def2‐TZVPD theory based on the LED decomposition scheme.

Nr.	Species	ΔE	DE	DE	DE	DE	Δ$E_{def.}^{geom.}$	${DE}_{def.}^{intra.}$
H_2_O			Na⋅⋅⋅FA	Na⋅⋅⋅H_2_O	FA⋅⋅⋅H_2_O	H_2_O⋅⋅⋅H_2_O		
0	neut.	−4.81	−64.52	–	–	–	0.35	59.71
	ion.	−28.01	−65.47	–	–	–	1.22	37.43
1	neut.	−17.97	−45.24	−82.45	−46.29	–	1.26	156.01
	ion.	−44.99	−57.70	−38.90	−12.18	–	0.76	70.09
2	neut.	−62.14	−191.60	−59.91	−163.50	+3.28	33.98	349.59
	ion.	−70.00	−54.27	−92.70	−4.51	+2.08	4.79	79.40
3	neut.	−75.12	−192.29	−70.30	−160.36	−57.80	32.43	389.93
	ion.	−81.87	−44.37	−110.70	−37.47	−3.57	1.06	114.24
4	neut.	−87.52	−157.52	−104.77	−235.36	−13.07	30.77	423.20
	ion.	−96.58	−44.42	−130.81	−33.86	−49.81	1.09	162.32
5	neut.	−101.64	−156.08	−117.17	−246.85	−64.66	31.22	483.12
	ion.	−112.95	−52.50	−115.57	−41.67	−159.57	2.48	256.36
6	neut.	−115.08	−151.61	−124.83	−281.29	−122.30	31.68	564.95
	ion.	−126.34	−41.83	−145.40	−54.65	−161.86	2.88	277.40
7	neut.	−127.31	−149.23	−128.75	−293.27	−169.01	32.03	629.09
	ion.	−139.35	−41.45	−147.72	−69.82	−206.06	3.62	325.70
8	neut.	−139.69	−146.93	−133.32	−298.92	−239.19	33.10	678.67
	ion.	−151.07	−36.93	−153.67	−85.99	−279.40	4.13	404.92

**Table 3:** The equilibrium geometry conformations obtained for the Na⋅⋅⋅FA⋅⋅⋅(H_2_O)_n_ and Na^+^⋅⋅⋅FA⋅⋅⋅ (H_2_O)_n_ (n=1–4) mixed clusters, based on energy (E_min_) or enthalpy (H_min_) minima, obtained at MN15/def2‐TZVP level of theory.

n	E_min_ for Na⋅⋅⋅FA⋅⋅⋅(H_2_O)_n_	E_min_ for Na^+^⋅⋅⋅FA⋅⋅⋅(H_2_O)_n_	H_min_ for Na^+^⋅⋅⋅FA⋅⋅⋅(H_2_O)_n_
	(*a*)	(*b*)	(*c*)
0	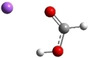	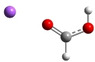	–
1	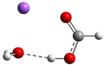	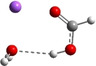	–
2	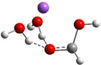	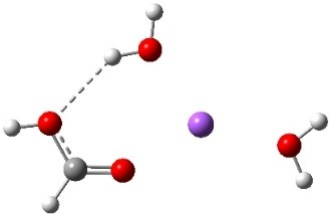	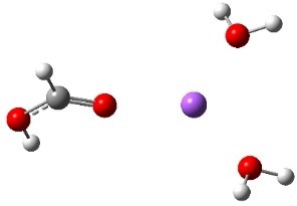
3	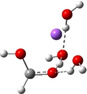	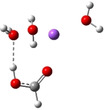	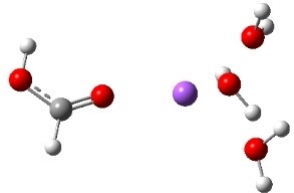
4	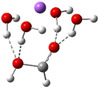	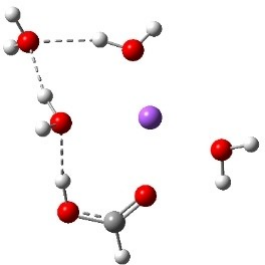	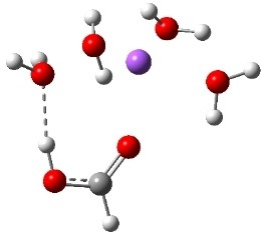

**Table 4:** The equilibrium geometry conformations found for the Na⋅⋅⋅FA⋅⋅⋅(H_2_O)_n_ and Na^+^⋅⋅⋅FA⋅⋅⋅(H_2_O)_n_ (n=5–8) mixed clusters, obtained at MN15/def2‐TZVP level of theory.

n	E_min_ for Na⋅⋅⋅FA⋅⋅⋅(H_2_O)_n_	E_min_ for Na^+^⋅⋅⋅FA⋅⋅⋅(H_2_O)_n_
	(*a*)	(*b*)
5	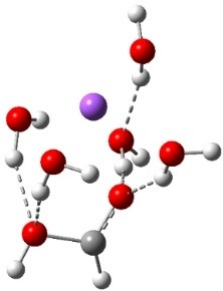	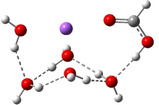
6	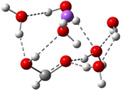	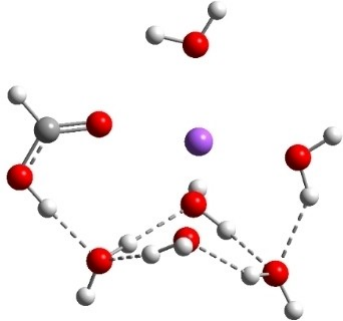
7	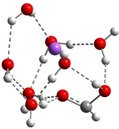	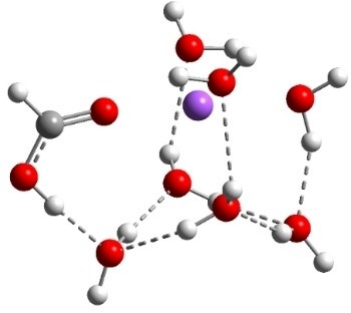
8	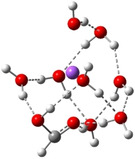	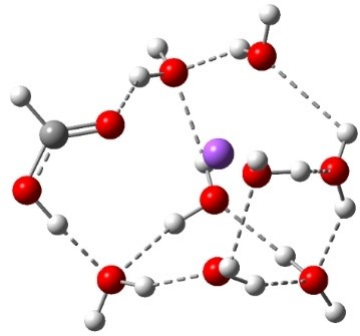

The simplest cluster aggregation is built by the binary form of the sodium ⋅⋅⋅ formic acid. In both, neutral and ionic cases the sodium atom is bounded to the oxygen atom of the FA (see Table 3: row n=0, columns a and b), where the Na⋅⋅⋅O distance is 2.397 Å for the neutral and 2.179 Å for the ionic case, respectively, while C=O bond distance of FA shows a relatively small change (from 1.208 Å in neutral form to 1.219 Å for ionic case). The corresponding ΔE value is −4.5 kcal/mol for the neutral and −26.8 kcal/mol for the ionic case, respectively. The relatively small cohesion of the neutral Na⋅⋅⋅FA complex is mainly given by the combined effect of two opposing interaction: attraction due to the electrostatic and charge polarization effects (−64.5 kcal/mol for neutral and −65.5 kcal/mol in the ionic case) and the excess energy to compensate orbital deformation of the fragments. Since the latter energy value (+37.4 kcal/mol for the ionic species vs +59.7 kcal/mol obtained for the neutral case) is much smaller for the ionic system, the strength of the interaction will be greater than the interaction energy obtained for the neutral system. The ionization energies defined in the first paragraph of the present section are the following: the VIE is 4.37 eV, AIE is 4.01 eV and AIE* is 4.03 eV. The cation relaxation energy (CRE) considered as the contribution of the orbital and geometry relaxation after the vertical ionization of an electron takes place is 0.36 eV. As regards the charge distribution, in the neutral case, there is a small amount of electron transfer (0.03e) from the FA to the Na atom (see Table 5). In the case of the ionic form, the vertical ionization acts exclusively on the Na atom and after the relaxation of the cation there is almost no electron transfer from the neutral FA to the ionized Na atom.

**Table 5:** The NBO fractional charge distributions of different cluster constituents computed for Na⋅⋅⋅FA⋅⋅⋅(H_2_O)_n_ (n=0–8) mixed clusters at MN15/def2‐TZVPD level of theory.

Nr. H_2_O	Species	q(Na)	q(FA)	q(H_2_O)
		[e]	[e]	[e]
0	neut.	0.03	−0.03	–
	ion.	1.00	0.00	–
1	neut.	0.14	−0.05	−0.09
	ion.	0.99	0.00	0.01
2	neut.	0.96	−0.87	−0.09
	ion.	0.98	0.01	0.01
3	neut.	0.94	−0.89	−0.05
	ion.	0.96	−0.01	0.05
4	neut.	0.95	−0.87	−0.08
	ion.	0.96	−0.01	0.05
5	neut.	0.94	−0.85	−0.09
	ion.	0.96	−0.02	0.06
6	neut.	0.93	−0.83	−0.10
	ion.	0.94	−0.02	0.08
7	neut.	0.91	−0.82	−0.09
	ion.	0.93	−0.03	0.10
8	neut.	0.90	−0.82	−0.08
	ion.	0.92	−0.03	0.11

Adding a water molecule to the Na⋅⋅⋅FA binary complex, its oxygen atom, as electron rich donor, forms a hydrogen bond (HB) with the OH fragment of the FA and as well as also strongly interacts with the sodium atom (see Table 3: row n=1, columns a and b). The cluster cohesion energy defined as the total intermolecular interaction energy between the three cluster components shows a significant energy increase. In the neutral electronic configuration, it is changed from −4.5 kcal/mol (the case of the Na⋅⋅⋅FA binary complex) to −16.7 kcal/mol when the water molecule was attached to this complex, while for the ionized electronic configuration the energy value increases to −44.2 kcal/mol. The energy decomposition shows a bit more complex picture. Namely, the pair‐interaction between the Na and FA components in the presence of the water molecule decreases compared with the Na⋅⋅⋅FA binary complex case and a strong Na⋅⋅⋅H_2_O interaction is formed. Similar strong interaction was observed between the FA and H_2_O cluster components, but only for the neutral electronic configuration. This fact looks interesting because the distance between Na and carbonyl oxygen for the neutral electronic configuration is reduced from 2.397 Å to 2.332 Å. At the same time, the C=O double bond of the FA is slightly enlarged in the neutral and is slightly diminished for the ionized case. All three: vertical, adiabatic energy and adiabatic enthalpy ionized potential decrease compared with the Na⋅⋅⋅FA binary complex case, while the magnitude of the CRE remains almost same. As regards the charge distribution, only for the neutral electronic configuration was found charge rearrangement. An amount of 0.14e move from the Na atom to FA (0.05e) and H_2_O (0.09e) cluster components (see Table 5).

The second water molecule induces structural changes in the Na⋅⋅⋅FA⋅⋅⋅(H_2_O)_2_ mixed cluster. In the neutral electronic configuration case, the unpaired 3s^1^ electron of the sodium leaves its parent atom and moves to the FA, inducing a charge separation inside the cluster. As a result, the geometry of the FA molecule also changes by transforming from the C=O double bond configuration of the carbonyl fragment to single C−O bond, and breaking the planar structure of FA due to the sp^3^ hybridization of the carbon (See Table 3: row n=2, columns a and b). Accordingly, the fractional charge of the sodium is 0.96e, while that of the FA is −0.87e. The charge difference of −0.09e is spread over the two water molecules. The C−O bond length becomes 1.297 Å, which clearly proves that it lost its double bond character. The Mayer's bond order[^17^, ^18^] value obtained for this type of C−O bond is 1.37, which shows that the newly formed bond loses its double bond character but is still stronger than the classical single bond. The total electronic interaction energy increases significantly (ΔE=−62.1 kcal/mol), however, more than a half of this binding energy is needed to compensate for the geometric deformation of the FA (${\Delta E}_{def.}^{geom}$ =+34.0 kcal/mol). As regards the pair‐interactions, due to the charge redistribution, the Na⋅⋅⋅FA interaction significantly increased (DE(Na⋅⋅⋅FA)=191.6 kcal/mol), but also the FA⋅⋅⋅H_2_O interaction (DE(FA⋅⋅⋅H_2_O)=−163.5 kcal/mol). Of course, all these strong pair‐interactions are partially neutralized by the large intra‐fragment electronic deformation energy of the cluster constituents. No such significant structural changes in the cluster geometry were observed in the ionized electronic case. Only the total intermolecular interaction energy increases, which is due to the increased interaction energy between Na^+^ ion and the two waters as compared with the previous cluster case having only one water molecule. Since, in the neutral case, significant changes were observed in both the geometric and the electron configuration structures, these effects also appear in the ionization potentials. Large CRE value of 2.09 eV, as the difference between the vertical and adiabatic ionization energies was found, which suggests that a significant rearrangement takes place in the electronic structure and geometry of the cluster after the ionization. On the other hand, the relatively high CRE value can be attributed to the increase in VIE as compared with the previous cluster case having only one water molecule, while the AIE changes only to a very small extent. One should also mention that in the ionized case, the geometry conformation with lowest enthalpy value at room temperature (see Table 3: row n=2, columns b and c) is not identical with the geometry conformation having the global energy minimum. One should also mention that several local minima geometries with higher conformational energy values were found for the neutral cluster case. Their geometries are presented in the in Figure S1 in SI file.

To ensure that the Na⋅⋅⋅FA⋅⋅⋅(H_2_O)_2_ mixed cluster is the critical cluster configuration where the energy driven geometry transition (changing the carbonyl fragment's C=O double bond to a weaker C−O bond having rather a single bond character) takes place, a new Na⋅⋅⋅FA⋅⋅⋅H_2_O mixed cluster having the starting geometry with the already distorted C−O bond was optimized. For this, specially prepared, geometry a stable configuration was found, but its conformational energy is higher with 2.5 kcal/mol than that of the cluster configuration with global energy minimum. On the other hand, specially prepared, starting geometry but for the Na⋅⋅⋅FA⋅⋅⋅(H_2_O)_2_ mixed cluster case, built based on the geometry with global energy minimum for the Na⋅⋅⋅FA⋅⋅⋅H_2_O mixed cluster having the C=O double bond configuration, was optimized without any geometrical constrain. The equilibrium geometry of this structure shows, that no conformational isomers where the carbonyl fragment's C=O keeps its double bond character was found.

The Na⋅⋅⋅FA⋅⋅⋅(H_2_O)_3_ mixed cluster already shows similar behavior as seen in the previous case. This system is also characterized by the broken planar symmetry as well as the single bond character of the C=O bond from the carbonyl group of the FA. For both the neutral and ionized cases, two as well as three equilibrium geometries were found. The conformational energy differences between the geometry isomers are relatively small, 0.6 kcal/mol in the neutral case as well as 0.4 kcal/mol and 1.4 kcal/mol for the ionized case. Overall, this cluster shows many similarities both for the neutral and ionized cases. The fractional charge distribution of the cluster components in neutral electronic configuration is 0.94e on the sodium atom, −0.89e on the FA and −0.05e, respectively, on the waters, while for the ionized case it is 0.96e on the sodium atom, −0.01e on the FA and 0.05e, respectively, on the waters. The total intermolecular interaction energy of the neutral system is −75.1 kcal/mol, where the dominant pair interaction is given by that found between the Na atom and the FA molecule (DE(Na⋅⋅⋅FA)=−192.3 kcal/mol), followed by the contribution between the FA molecule and waters (DE(FA⋅⋅⋅H_2_O)=−160.4 kcal/mol) and by the interaction between Na atom and the water molecules (DE(Na⋅⋅⋅H_2_O)=−70.3 kcal/mol). The smallest contribution is obtained for the water‐water interaction, which, compared with the previous cluster case, has an attractive character (DE(H_2_O⋅⋅⋅H_2_O)=−57.8 kcal/mol). In the case of the ionized system, the total intermolecular interaction energy is −75.6 kcal/mol, which mainly comes from the interaction between the Na^+^ and waters (DE(Na^+^⋅⋅⋅H_2_O)=−110.7 kcal/mol), while the contribution between Na^+^ and FA is −44.4 kcal/mol and that of between FA and water is −37.5 kcal/mol. The water‐water interaction is almost insignificant. All these strong attractive interactions between different cluster components are strongly screened by strong electronic and geometry deformation energy values of the cluster components. As regards the different ionization potentials, the VIE becomes a bit larger than in the previous case, while AIP and AIP* a bit smaller. Of course, these changes further increase the amount of the cation relaxation energy (CRE=2.29 eV) for this cluster case. Similarly, as it was found for the Na^+^⋅⋅⋅FA⋅⋅⋅(H_2_O)_2_ mixed cluster case, the geometry conformation with the lowest enthalpy value at room temperature (see Table 3: row n=3, columns b and c) is not identical with the geometry conformation having the global energy minimum. Also, for this configuration several local minima geometries with higher conformational energy values were found for the neutral cluster case. Their geometries are presented in the in Figure S1 in SI file.

From the larger clusters point of view, the neutral species of the Na⋅⋅⋅FA⋅⋅⋅(H_2_O)_4_ mixed cluster can be considered as a reference (or core) cluster configuration, as this cluster will also occurs as a subsystem in the case of larger clusters. A number of 5 different cluster conformations for the neutral electronic case and 18 different cluster conformations for the ionized case were found. The neutral cluster with the global energy minimum shows a V‐shaped form where its edge is given by the oxygen atoms of the FA molecule. In addition, two waters are bound to each oxygen of the FA and so the four waters with their oxygen atoms make a ring around the sodium (See Table 3: row n=4, column a). In the ionized case, apart from the cluster structures with up to three water molecules, a water molecule that does not bind directly to the ionized Na atom also appears. It forms strong HBs with other waters which are in direct contact with the Na ion and of which electron clouds are strongly polarized by the ion. The global equilibrium geometry of the neutral species keeps the previously observed broken double bond character, the charge distribution and the characteristic bond distances are very similar with the n=2,3 neutral cluster cases. The total energy of the intermolecular interaction of the neutral form increases (ΔE=−87.5 kcal/mol), but interesting rearrangements can be observed. Namely, the Na⋅⋅⋅FA interaction, compared with the Na⋅⋅⋅FA⋅⋅⋅(H_2_O)_3_ case, decreases and is no longer the most dominant pair interaction (DE(Na⋅⋅⋅FA)=−157.5 kcal/mol). Instead, an increased FA⋅⋅⋅H_2_O interaction can be observed (DE(FA⋅⋅⋅H_2_O)=−235.4 kcal/mol). The total intermolecular interaction for the ionized species is −96.6 kcal/mol and compared with the previous ionized cluster case, increasing contributions for the pair interactions were found for the Na^+^⋅⋅⋅H_2_O and H_2_O⋅⋅⋅H_2_O type interactions. Again, for the ionized species it was found that, the geometry conformation with the lowest enthalpy value at room temperature (see Table 3: row n=4, columns b and c) is not identical with the geometry conformation having the global energy minimum. In general, it can be observed that in the case of the total electronic energy at 0 K the largest the number of HBs in the cluster the lowest conformational energy is obtained. Opposite effect can be observed for the enthalpy where the thermal effects of the vibrations are included. Here, at room temperature, smaller number of HBs in the cluster gives smaller thermal effects and thus lower enthalpy values. The VIE increases a bit further (from 5.88 eV for n=3 to 5.99 eV for n=4), while the AIE and AIE* values remain almost the same as one was found for n=3 case.

The case of Na⋅⋅⋅FA⋅⋅⋅(H_2_O)_5_ mixed cluster is, again, a reference (or core) cluster configuration, but this time for the ionized species. The cyclic water tetramer with tetrahedron form binds, on one hand, the FA molecule through the OH fragment of the FA, and on the other hand, the Na^+^ ion by the two opposite waters from cyclic configuration. The fifth water is located on the opposite side of Na^+^ relative to the FA molecule (see Table 4: row n=5, column b). As regards the total interaction energy, in the case neutral electronic configuration it was obtained −101.6 kcal/mol, while for the ionized case −113.0 kcal/mol. If one analyses the pair interactions, one can observe that for the neutral species the Na⋅⋅⋅FA interaction remains almost the same (DE(Na⋅⋅⋅FA)=−156.1 kcal/mol) while those interactions where the waters are involved increase (DE(Na⋅⋅⋅H_2_O)=−117.2 kcal/mol, DE(FA⋅⋅⋅H_2_O)=−246.9 kcal/mol and DE(H_2_O⋅⋅⋅H_2_O)=−64.7 kcal/mol). So far, we have found that in the ionized case, the Na⋅⋅⋅FA pair interaction decreases as the number of water molecules increases within the cluster. However, the n=5 case differs from this usual trend, as the strength of this pair of interactions (DE(Na⋅⋅⋅FA)_n=5_=−52.5 kcal/mol) increases again compared to the n=4 case (DE(Na⋅⋅⋅FA)_n=4_=−44.4 kcal/mol). This different behavior can also be explained by the fact that the d(Na⋅⋅⋅O) distance decreases to 2.233 Å. At this time, cluster configurations for which the geometry of the global energy minimum is not the same with the geometry of the lowest enthalpy value at room temperature are no longer found. The VIE shows the largest value (6.05 eV) so far, while the results for AIE and AIE* decrease 0.1 eV as compared with the n=4 case. Consequently, the cluster relaxation energy further increases to 2.58 eV.

No significant differences were found for Na⋅⋅⋅FA⋅⋅⋅(H_2_O)_n_ (n=6,8) mixed cluster cases. Based on the characteristic configurations found for n=4 in neutral and n=5 in ionized cases, the one up to three waters increasingly cover the Na atom, which is almost completely enclosed in the case of n=8 water and formic acid (see Table 4: rows n=5–8, columns a and b). The d(Na⋅⋅⋅O) and d(C=O) distances are enlarged evenly, both for neutral and ionized electronic configurations. Namely, the d(Na⋅⋅⋅O) distances in neutral case are 2.381 Å for n=6, 2.404 Å for n=7 and 2.423 Å for n=8, respectively. Similarly, the total interaction energy shows a steady increase for both electronic configurations. The ΔE values for neutral case are −115.1 kcal/mol for n=6, −127.3 kcal/mol for n=7 and −139.7 kcal/mol for n=8, respectively, while those for the ionized case are −126.3 kcal/mol for n=6, −139.4 kcal/mol for n=7 and −151.1 kcal/mol for n=8, respectively. In the case of pair interactions, it was found that DE(Na⋅⋅⋅FA) decreases slightly, the magnitude of the DE(Na⋅⋅⋅H_2_O) and DE(FA⋅⋅⋅H_2_O) interactions increases somewhat more strongly, while that of DE(H_2_O⋅⋅⋅H_2_O) increases much faster as the value of n increases. The trend is similar for the solvated electron. The Na atom is in an almost fully ionized state, while most of the charge that left the Na atom is localized on the formic acid, only a small amount of them migrate to the water molecules. No significant change was observed for the different ionization potentials either. While the VIE values show a saturation effect, a slightly perceptible decrease can be seen for the AIE and AIE* values.

**Figure 3:**
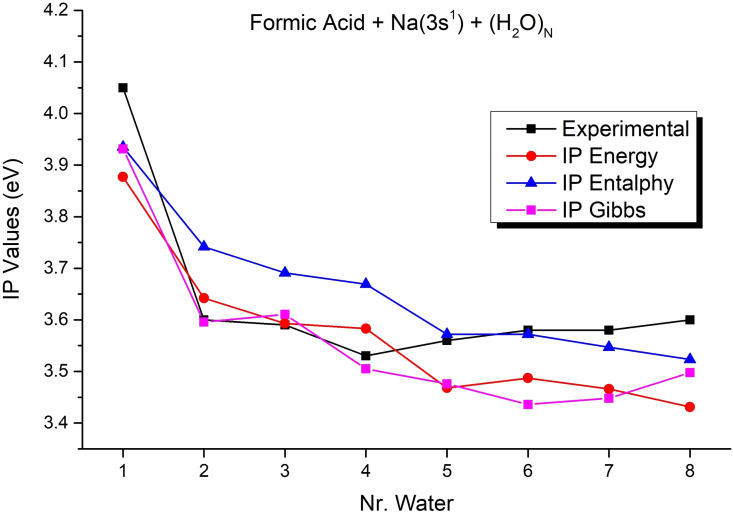
Calculated Ionization Potentials including energy, enthalpy and entropy effects. The experimental values are the appearance energy of Na−FA(H_2_O)_n_ clusters. Each value is taken at 10 % of the maximum signal detected in the sampled wavelength range.

If one interprets the results in a larger framework, it can be seen that n=2 is the critical geometry where the energy required for the rearrangement of the electronic structure (the transfer of the unpaired 3s^1^ electron of the sodium atom to the FA molecule) can be overcame by the contribution of the interaction energies formed between Na^+^⋅⋅⋅H_2_O and FA^−^⋅⋅⋅H_2_O. The FA molecule with the excess electron loses its original molecular structure, the C=O bond becomes longer (from 1.213 Å to 1.297 Å) and its bond order decreases from 1.85 to 1.37, as well as it also loses its planar structure. These changes in charge distribution and in geometry conformation make it possible for a significant rearrangement inside the cluster after the ionization. This also means a large, unusual cluster relaxation energy (larger than 2 eV) and thus large difference between the vertical and adiabatic ionization potentials. The enthalpy effect (the thermal effects of the vibrations) in the adiabatic ionization energy is 0.1 eV in average and fits very well with the experimentally measured values (see Figure 3), while the slightly increase of the adiabatic ionization energy values for n larger than six water molecules is due to the entropic effects.

In order to understand more accurately the structural topology of Na⋅⋅⋅FA⋅⋅⋅(H_2_O)_n_ (n=1–8) mixed clusters in either neutral or ionized form, it is worth examining briefly the structures of the original FA⋅⋅⋅(H_2_O)_n_ (n=1–8) mixed clusters. Accordingly, the equilibrium structures of global geometries for FA⋅⋅⋅(H_2_O)_n_ (n=1–8) mixed clusters were computed at the same MN15/def2‐TZVPD level of theory, while the decomposition of the intermolecular interaction energies were performed in the same framework of the DLPNO‐CCSD(T)/def2‐TZVPD theory. The geometrical arrangements of different cluster configurations are presented in the in Figure S2 in SI file, while the total interaction energies and pair‐interaction energies between different cluster components are given in Table 6. As for the interactions between the cluster components, it can be observed that for cases larger than n=4 there is no longer a substantial increase in the interaction between FA and waters, but rather an oscillating behavior. While the water⋅⋅⋅water interaction shows a significant increase as the number of water molecules increases in the cluster. If, on the other hand, the FA⋅⋅⋅(H_2_O)_n_ and homogeneous water clusters are compared with the Na‐doped Na⋅⋅⋅FA⋅⋅⋅(H_2_O)_n_ (n=1–8) mixed cluster systems, we can see that the presence of the Na atom thoroughly rearranges the cluster geometries, in which the sodium behaves like a nucleus around which the FA and water molecules are arranged.

**Table 6:** The total interaction energies (ΔE in kcal/mol) and interaction energy components between different cluster constituents (ΔE in kcal/mol) for the neutral FA⋅⋅⋅(H_2_O)_n_ (*n*=1–8) mixed clusters obtained at DLPNO‐CCSD(T)/def2‐TZVPD theory based on the supramolecular decomposition scheme.

Nr. H_2_O	ΔE	DE FA⋅⋅⋅H_2_O	DE H_2_O⋅⋅⋅H_2_O	${\Delta E}_{def.}^{geom.}$
1	−10.40	−10.40	–	0.51
2	−22.11	−18.29	−3.82	2.13
3	−31.20	−21.46	−9.74	2.53
4	−42.79	−24.60	−18.19	3.78
5	−51.92	−26.40	−25.52	4.21
6	−62.58	−25.28	−37.30	4.58
7	−72.87	−28.51	−44.36	5.72
8	−81.65	−21.01	−60.64	4.30

### The Electronic Excitation and Excited State Relaxation

As one can see from Table 1, the vertical ionization potentials of different sized Na⋅⋅⋅FA⋅⋅⋅(H_2_O)_n_ (n=3–8) mixed clusters show values above 5 eV, while the photon energy which induces the ionization lies under 4 eV. Apparently, this amount of energy would not be sufficient for directly ionizing an electron of the cluster and one of the possible explanations would be the autoionization over the Rydberg excited states[^19^, ^20^, ^21^] during the orbital relaxation. Accordingly, the low lying electronic excited states of each Na⋅⋅⋅FA⋅⋅⋅(H_2_O)_n_ (n=1–8) cluster configuration were computed considering the TD‐DFT framework by using the ωB2PLYP range‐separated double‐hybrid XC functional and def2‐TZVPD basis set. The first six electronic excited state energies for different sized Na⋅⋅⋅FA⋅⋅⋅(H_2_O)_n_ (n=1–8) mixed clusters are collected in Table 7. The theoretical UV‐Vis absorption spectra for different sized Na⋅⋅⋅FA⋅⋅⋅(H_2_O)_n_ (n=1–8) mixed clusters are presented in Figure S3 in SI file and the shapes of natural difference orbitals (NDO) defined as the difference density between ground and excited states are given in Figure S4 of the SI file. The experimental results showed that there is a minimum value in the excitation energy (at around 3.5 eV) of the laser below which the mixed clusters do not show any ionization effects.

**Table 7:** The first six (S_1_–S_6_) electronic excited state energies (in eV) computed for Na⋅⋅⋅FA⋅⋅⋅(H_2_O)_n_ (n=0–8) mixed clusters at ωB2PLYP/def2‐TZVPD level of theory.

Nr. H_2_O	S_1_	S_2_	S_3_	S_4_	S_5_	S_6_
0	1.59	1.77	2.44	2.50	3.22	4.26
1	1.52	1.56	2.31	2.77	3.19	4.20
2	2.92	3.90	4.09	4.46	5.50	5.59
3	3.11	4.16	4.39	4.54	5.55	5.56
4	3.39	4.18	4.85	4.99	5.46	5.67
5	3.54	4.39	4.99	5.16	5.60	5.68
6	3.56	4.60	4.82	5.25	5.74	5.88
7	3.62	4.70	4.86	5.39	5.79	5.89
8	3.62	4.63	4.86	5.34	5.76	5.90

On the other hand, if one analyses the profiles of the NDOs, it can be seen that the first six electron excitations, without exception, are all Rydberg‐type transitions (See Figure S4 of the SI file). For the Na⋅⋅⋅FA⋅⋅⋅(H_2_O)_n_ (n=0,1) mixed cluster cases, the “negative” (or electron hole) densities (orange color) are mainly spread over at the vicinity of the Na atom, while the “positive” (or excited electron) densities (blue color) either spread far from any atoms of the cluster (case of S_1_–S_3_ excited states) or are located around the FA or H_2_O cluster constituents. For the Na⋅⋅⋅FA⋅⋅⋅(H_2_O)_n_ (n=2–8) mixed cluster cases, since the electron transfer from Na to FA already occurred at the ground state level, the electronic excitation promotes electron transitions from the FA either to the waters or to the empty space around the waters. In these cases, the only differences can be observed between S_1_ states and the other excited states. This is manifested in the fact that while in the case of the S_1_ states the excitation is localized to a compact part of the space, in the case of the higher excited states this is divided into several separate space parts defined by different Rydberg orbitals.

## Conclusions

We carried out a study on FA(H_2_O)_n_ clusters using the experimental method of Na‐doping of the clusters produced in the supersonic expansion of vapors of aqueous solutions with low concentration of FA. The mass spectra were considerably simplified as mixed clusters FA_n_(H_2_O)_m_ were not detected although the clusters of interest were found at low intensity. This allowed to reason on mass spectrometry and ionization energies data fundamentally unaffected, or not very affected, by spurious fragmentation contributions.

From mass spectra, it results doped mixed acid‐water clusters have increased peak intensity at larger FA(H_2_O)_n_ clusters with respect to the water cluster mass distribution. This could indicate a role of the acid in the water aggregations, at least on small clusters, and for the expansion conditions used for the experiment. In particular, the increased peak intensity at larger Na−FA(H_2_O)_n_ with respect to Na−(H_2_O)_n_ could indicate water molecules aggregate around small FA(H_2_O)_n_ cluster more than around (H_2_O)_n_ clusters. The appearance energy values of mixed doped clusters were also determined and found to drop from 4 eV (n=1) to 3.5–3.6 eV for clusters containing n≥2 water molecules.

Theoretical calculations have shown that neutral and ionized sodium‐doped clusters are very stable with a fundamental role played by the sodium in rearranging the geometry of the cluster already formed. The adiabatic ionization energies calculated with the DFT are in agreement with the measured appearance energies. This may suggest a contribution in the ion signals from autoionization phenomena during orbital relaxing of Rydberg‐type excited states although fragmentation contribution from unidentified cluster classes cannot be ruled out. Specifically, theoretical calculations on the low lying electronic excited states of each Na⋅⋅⋅FA⋅⋅⋅(H_2_O)_n_ (n=1–8) show that the states S_2_ (for n=2, 3) and S_1_ (for n=4–8) electronic excited states are close to the appearance energies. In addition, the profiles of the NDOs, computed for the first six electron excitations, indicate Rydberg‐type transitions. A thorough understanding of relaxation channels from these exited states, which could eventually lead to ionization, is beyond the scope of this paper. Considering our results so far, the autoionization phenomena cannot be ruled out in these sodium‐doped clusters. The existence of the Rydberg‐type electronic excitations and implicitly the presence of electron orbitals spreading strongly in the intermolecular space may also explain the possibility that the excited electron can move away from the cluster core, formed by the sodium atom and the formic acid, and even can leave the cluster, leaving it in an ionized form. This hypothesis of electron migration at large distances inside the cluster is also supported by the study in ref [22] showing that an extra electron attached to neutral water cluster can mainly be located at the outer surface of the cluster. So, it is very plausible that the clusters with lower energy structures calculated with the DFT are the ones that actually contribute to the ionization signals.

Finally, theoretical calculations on neutral FA(H_2_O)_n_ clusters have shown that, in the low energy geometric configurations, small FA‐Water clusters (n=1–8) are very stable. By considering the pair‐interaction energies between different cluster components, FA‐Water interaction results stronger than water‐water interaction for n=1–4, indicating that water molecules aggregate preferentially with acid molecule mainly in the first stage of cluster formation. Afterward this interaction stabilizes while water‐water interaction increased.

Further investigations need to definitively quantify the influence of the simplest carboxylic acid on water aggregations. Our results indicate FA surely affects the formation of the molecular aggregate in the initial stage of the process.

## Experimental Section

The experimental setup is described in details in.[^13^, ^14^, ^23^] Basically, it was constituted by a molecular beam, a homemade Wiley–McLaren time of flight spectrometer (TOF), and an Optical Parametric Oscillator (OPO) laser source. The molecular beam was produced by the supersonic expansion of vapours from 0.02 M formic acid aqueous solution (Aldrich) seeded in Helium at 6 bar stagnation pressure in the vacuum by means of a pulsed valve (Parker mod 99, 180 μs pulse duration, 0.8 mm nozzle diameter). The supersonic jet was selected by a 1 mm conical skimmer (Beam Dynamics), 2 cm away from the valve nozzle, and sent to a small stainless‐steel oven, kept at 200 °C, which vaporized sodium flakes. The Na atoms produced in the oven picked up the clusters. Through a 3 mm hole, the doped clusters entered the 200 V/cm extraction region of the TOF.^[23]^ The TOF axis was collinear with the molecular beam and the mass resolution was m/Δm≈500@m/z=83. The OPO laser source (Opotek, RADIANT 355, peak energy 30 mJ @400 nm, 4 ns pulse duration, linewidth 4–9 cm^−1^) was equipped with doubling and mixing non‐linear crystals to get 410–2500 nm tuning range. The wavelength range used for the presented measurements was 280–370 nm. The laser energy was measured with a pyroelectric energy sensor (Gentec‐EO mod. QE12LP‐S‐MB‐C0). During the measurements, the laser energy was maintained below 0.1 mJ in the sampled wavelength range.

Mass spectra were acquired using DAQ card (National Instruments PCI 5152) and driven by LabVIEW® program. The mass spectra were background subtracted and acquired by accumulating 1000 ionization events. The photoionization efficiency spectra were obtained by integrating the mass peak signals acquired by varying the laser energy with a 2 nm wavelength step.

### Computational Details

The equilibrium geometries and thermodynamic potentials of the neutral and ionized species of the Na⋅⋅⋅FA⋅⋅⋅(H_2_O)_n_ (n=1–8) mixed clusters were obtained in the framework of the Density Functional Theory (DFT) implemented in the PSI4 code.^[24]^ Accordingly, the MN15^[25]^ exchange‐correlation functional together with the def2‐TZVPD[^26^, ^27^] basis set were employed for the electronic structure calculations. The vertical‐ (VIE) and adiabatic ionization (AIE) energies were computed considering the total energy differences between the neutral (E(N)) and its cationic (E(N‐1)) forms. In order to include the thermal effects in the adiabatic ionization energy calculation, the enthalpy and Gibbs free energy values for the neutral and ionic species were also computed via the thermochemistry module of the vibrational normal mode analysis. The intermolecular interaction energy decomposition was performed using the local energy decomposition (LED) method[^28^, ^29^] by decomposing the DLPNO‐CCSD(T) (domain based local pair natural orbital coupled‐cluster method with single, double and perturbative triple excitations) energy[^30^, ^31^] into physically meaningful contributions as is implemented in the ORCA code[^32^, ^33^] including the RIJCOSX approximation^[34]^ and the Def2/J^[35]^ and def2‐TZVPP/C^[36]^ auxiliary basis sets. The electron population analysis was carried out with the Natural Bond Orbital (NBO) theory[^37^, ^38^] as performed with NBO 5.9 program.^[39]^ The electronic excited states were computed using the time‐dependent DFT (TD‐DFT) method considering the ωB2PLYP^[40]^ range‐separated double‐hybrid exchange‐correlation (XC) functional implemented in the ORCA[^32^, ^33^] code. The ωB2PLYP XC functional has shown improved performance not only for long‐range excitations (Rydberg and charge‐transfer excitations) but also for local‐valence excitations and the difficult to treat first two excitations in polycyclic aromatic hydrocarbons.^[40]^ The molecular graphics (figures) were drawn using the avogadro ^[41]^ and gaussview ^[42]^ molecular editor and visualizer softwares.

## Conflict of interest

The authors declare no conflict of interest.

1

## Supporting information

As a service to our authors and readers, this journal provides supporting information supplied by the authors. Such materials are peer reviewed and may be re‐organized for online delivery, but are not copy‐edited or typeset. Technical support issues arising from supporting information (other than missing files) should be addressed to the authors.

Supporting Information

## Data Availability

The data that support the findings of this study are available from the corresponding author upon reasonable request.
