# Supplementary material for: Photoionization, Structures, and Energetics of Na‐Doped Formic Acid–Water Clusters
Source: Chemphyschem. 2022 Jan 27;23(5):e202100861. doi: 10.1002/cphc.202100861 (PMC9303463; doi:10.1002/cphc.202100861)
Supplement: Supplementary file 1 — Supporting Information [file CPHC-23-0-s001.pdf]

ChemPhysChem

Supporting Information

# Photoionization, Structures, and Energetics of Na-Doped Formic Acid–Water Clusters

Attila Bende, Maria F. Gaele, and Tonia M. Di Palma*

# Supporting Information

| **Table of Contents** | **Pages** |
|---|---|
| Computation information and geometries of Sodium – Formic Acid – Water mixed cluster | 2 |
| **Figure S1.** The geometry conformations of different local minima found for Na···FA ···$(H_2O)_n$ (n = 2,3) neutral mixed clusters, obtained at MN15/def2-TZVP level of theory. | 3 |
| **Table S1**. The total intermolecular interaction energies (ΔE), enthalpies (ΔH) and Gibbs free energies (ΔG) of the Na···FA···$(H_2O)_n$ (*n* = 0-8) mixed clusters obtained at MN15/def2-TZVPD level of theory and T = 298.15 K. | 3 |
| **Figures S2.** The equilibrium geometry conformations found for the FA···$(H_2O)_n$ (n = 1,8) mixed clusters, obtained at MN15/def2-TZVP level of theory. | 4 |
| **Figure S3**. The theoretical UV-Vis absorption spectra computed for Na···FA···$(H_2O)_n$ (n = 0,8) mixed clusters computed at ωB2PLYP/def2-TZVPD level of theory. | 5 |
| **Figure S4**. The natural difference orbital (NDO) profiles compiled for the first six electronic excited states of Na···FA···$(H_2O)_n$ (n = 0,8) mixed clusters obtained at ωB2PLYP/def2-TZVPD level of theory. | 6 – 10 |
| **Figure S5 a)**, **b).** Expanded mass spectra | 11-12 |

**<u>Molecular Formula</u>**: Formic Acid – (**FA**); Sodium – Formic Acid – Water mixed cluster: **Na**···**FA**···(**$H_2O$**)$_n$ (n = 1,8)

**Type of calculation**: Geometry optimization of equilibrium geometries for the neutral and the singly ionized electron configurations of Na···FA···($H_2O$)$_n$ and $Na^+$···FA···($H_2O$)$_n$ (n = 1,8) mixed clusters; Theoretical UV-Vis absorption spectra; Natural difference orbitals between the ground and the given excited state; Conical intersection between the ground and first excited states.

**Software**: PSI4 [1], ORCA 4.2.1 [2,3]

**Level of Theories**: DFT with M15 [4] exchange-correlation functional implemented in PSI4 as well as DFT with wB2PLYP [5] exchange-correlation functional and pair natural orbital based local coupled cluster (DLPNO-CCSD(T)) [6,7] methods implemented in ORCA.

**Basis Set**: def2-TZVPD [8,9]

**References**:


[1] R. M. Parrish, L. A. Burns, D. G. A. Smith, A. C. Simmonett, A. E. DePrince III, E. G. Hohenstein, U. Bozkaya, A. Yu. Sokolov, R. Di Remigio, R. M. Richard, J. F. Gonthier, A. M. James, H. R. McAlexander, A. Kumar, M. Saitow, X. Wang, B. P. Pritchard, P. Verma, H. F. Schaefer III, K. Patkowski, R. A. King, E. F. Valeev, F. A. Evangelista, J. M. Turney, T. D. Crawford, and C. D. Sherrill, "Psi4 1.3.2: An Open-Source Electronic Structure Program Emphasizing Automation, Advanced Libraries, and Interoperability", *J. Chem. Theory Comput.*, **13**(7) 3185–3197 (**2017**).

[2] F. Neese, "The ORCA program system", *WIRES Comput. Mol. Sci.* **2**(1), 73, (**2012**).

[3] F. Neese, "Software update: the ORCA program system, version 4.0", *WIRES Comput. Mol. Sci.* **8**(1), e1327, (**2017**).

[4] H. S. Yu, X. He, S. L. Li and D. G. Truhlar, "MN15: A Kohn–Sham global-hybrid exchange–correlation density functional with broad accuracy for multi-reference and single-reference systems and noncovalent interactions", *Chem. Sci.*, **7**, 5032–5051 (**2016**).

[5] M. Casanova-Páez, M. B. Dardis, and L. Goerigk, "ωB2PLYP and ωB2GPPLYP: The First Two Double-Hybrid Density Functionals with Long-Range Correction Optimized for Excitation Energies", *J. Chem. Theory Comput.* **15**(9), 4735, (**2019**).

[6] C. Riplinger and F. Neese, "An efficient and near linear scaling pair natural orbital based local coupled-cluster method.", *J. Chem. Phys.*, **138**, 034106 (**2013**).

[7] M. Saitow, U. Becker, C. Riplinger, E.F. Valeev and F. Neese, "A new linear scaling, efficient and accurate, open-shell domain-based pair natural orbital coupled cluster singles and doubles theory.", *J. Chem. Phys.*, **146**, 164105 (**2016**).

[8] F. Weigend and R. Ahlrichs, "Balanced basis sets of split valence, triple zeta valence and quadruple zeta valence quality for H to Rn: Design and assessment of accuracy", *Phys. Chem. Chem. Phys.* **7**, 3297 (**2005**).

[9] D. Rappoport and F. Furche, "Property-optimized Gaussian basis sets for molecular response calculations", *J. Chem. Phys.* **133**, 134105 (**2010**).

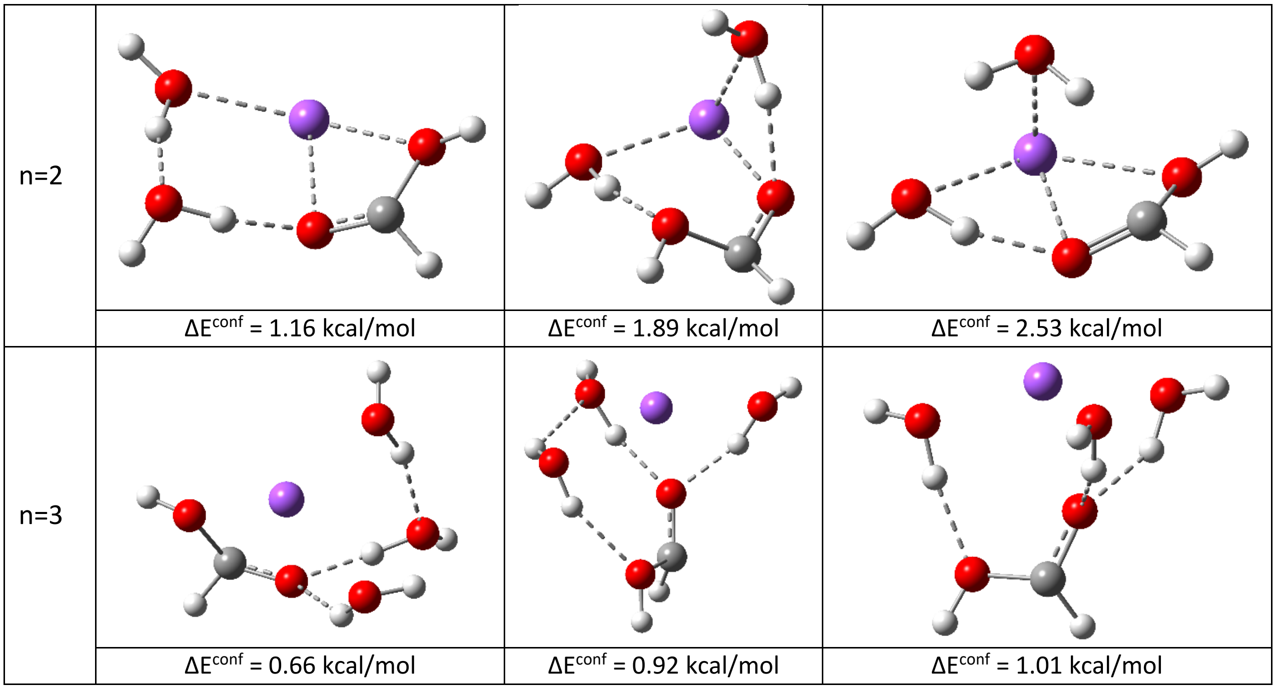


**Figure S1**. The equilibrium geometry conformations of different local minima found for the Na···FA···$(H_2O)_n$ (n = 2,3) neutral mixed clusters, obtained at MN15/def2-TZVP level of theory.

**Table S1**. The total intermolecular interaction energies (ΔE), enthalpies (ΔH) and Gibbs free energies (ΔG) of the Na···FA···$(H_2O)_n$ ($n$ = 0-8) mixed clusters obtained at MN15/def2-TZVPD level of theory and T = 298.15 K. All values are given in kcal/mol.

| Nr. $H_2O$ | Species | ΔE | ΔH | ΔG |
|---|---|---|---|---|
| 0 | Neut. | -4.83 | -5.63 | 0.31 |
| | Ion. | -28.81 | -27.08 | -20.02 |
| 1 | Neut. | -19.13 | -19.05 | -2.98 |
| | Ion. | -44.19 | -42.75 | -26.78 |
| 2 | Neut. | -31.99 | -31.30 | -5.60 |
| | Ion. | -64.35 | -61.71 | -39.07 |
| 3 | Neut. | -48.12 | -45.86 | -11.05 |
| | Ion. | -79.74 | -75.21 | -42.25 |
| 4 | Neut. | -62.74 | -58.48 | -11.55 |
| | Ion. | -94.92 | -88.62 | -45.19 |
| 5 | Neut. | -76.01 | -70.24 | -13.79 |
| | Ion. | -110.54 | -102.34 | -45.59 |
| 6 | Neut. | -89.48 | -81.89 | -14.08 |
| | Ion. | -123.54 | -113.97 | -49.31 |
| 7 | Neut. | -101.89 | -92.55 | -14.58 |
| | Ion. | -136.43 | -125.22 | -49.53 |
| 8 | Neut. | -113.41 | -102.04 | -15.11 |
| | Ion. | -148.60 | -135.61 | -48.92 |

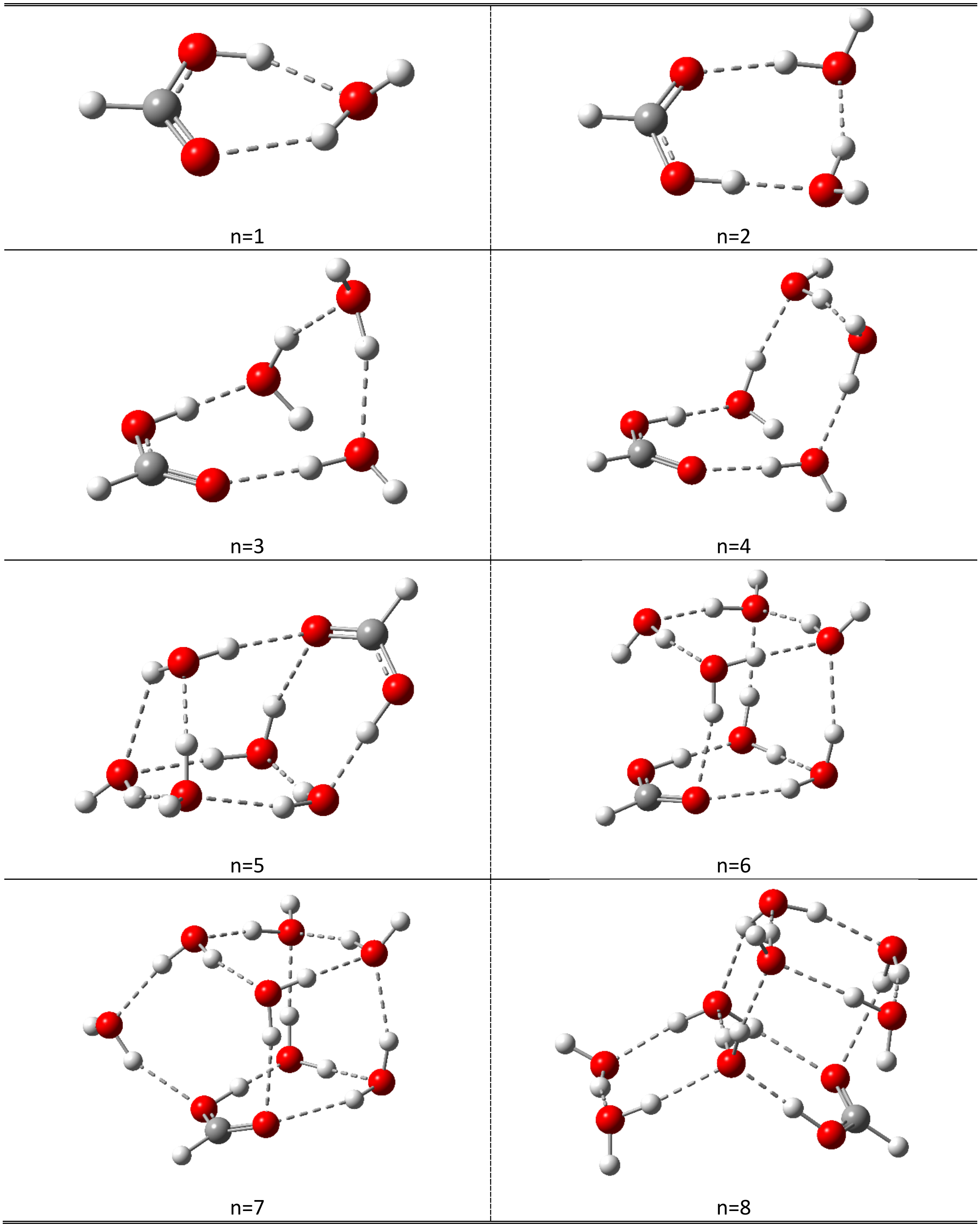


**Figure S2**. The equilibrium geometry conformations found for the FA···$(H_2O)_n$ (n = 1,8) mixed clusters, obtained at MN15/def2-TZVP level of theory.

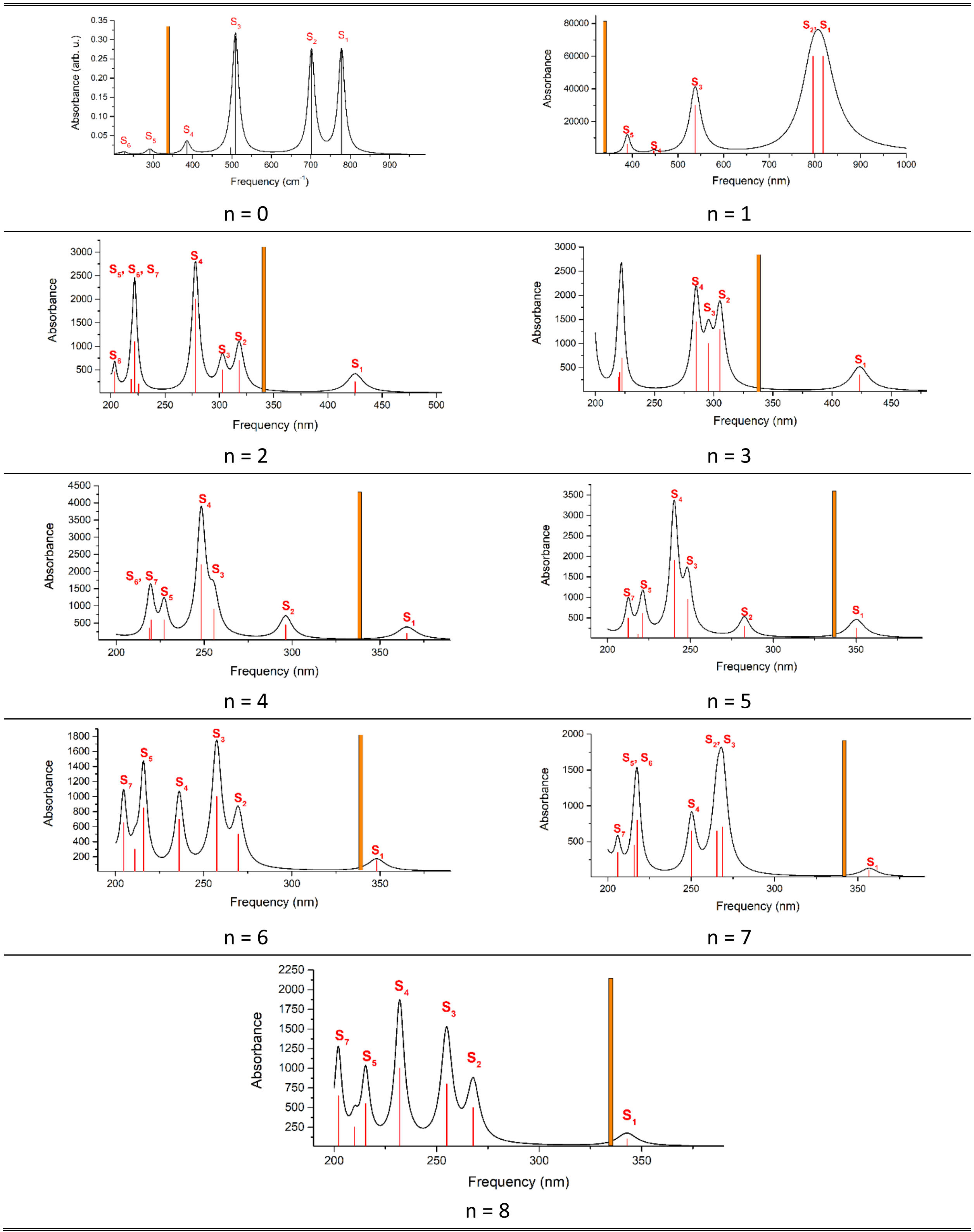


**Figure S3**. The theoretical UV-Vis absorption spectra computed for Na···FA···$(H_2O)_n$ (n = 0,8) mixed clusters computed at ωB2PLYP/def2-TZVPD level of theory.

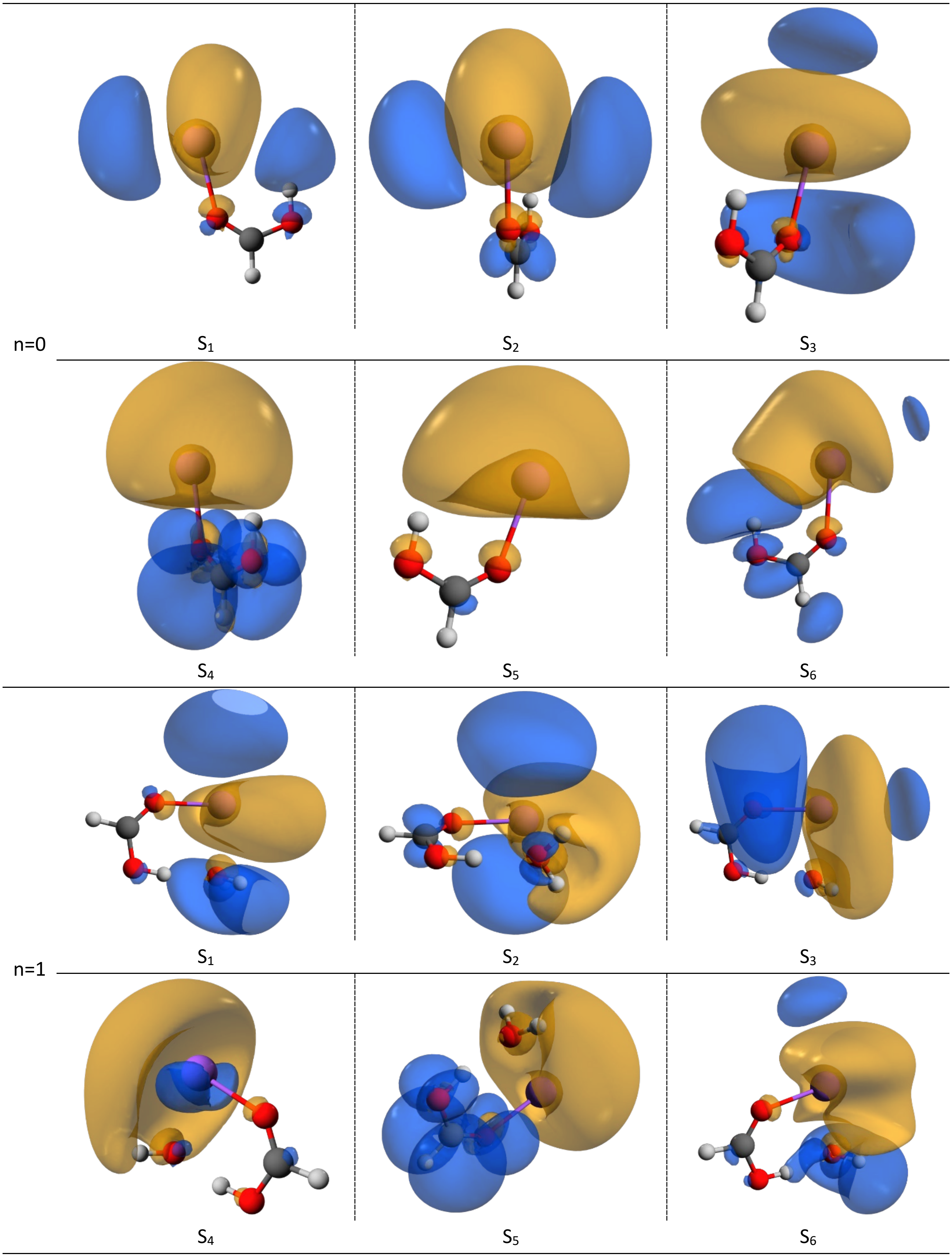
n=0
S1
S2
S3
S4
S5
S6
n=1
S1
S2
S3
S4
S5
S6

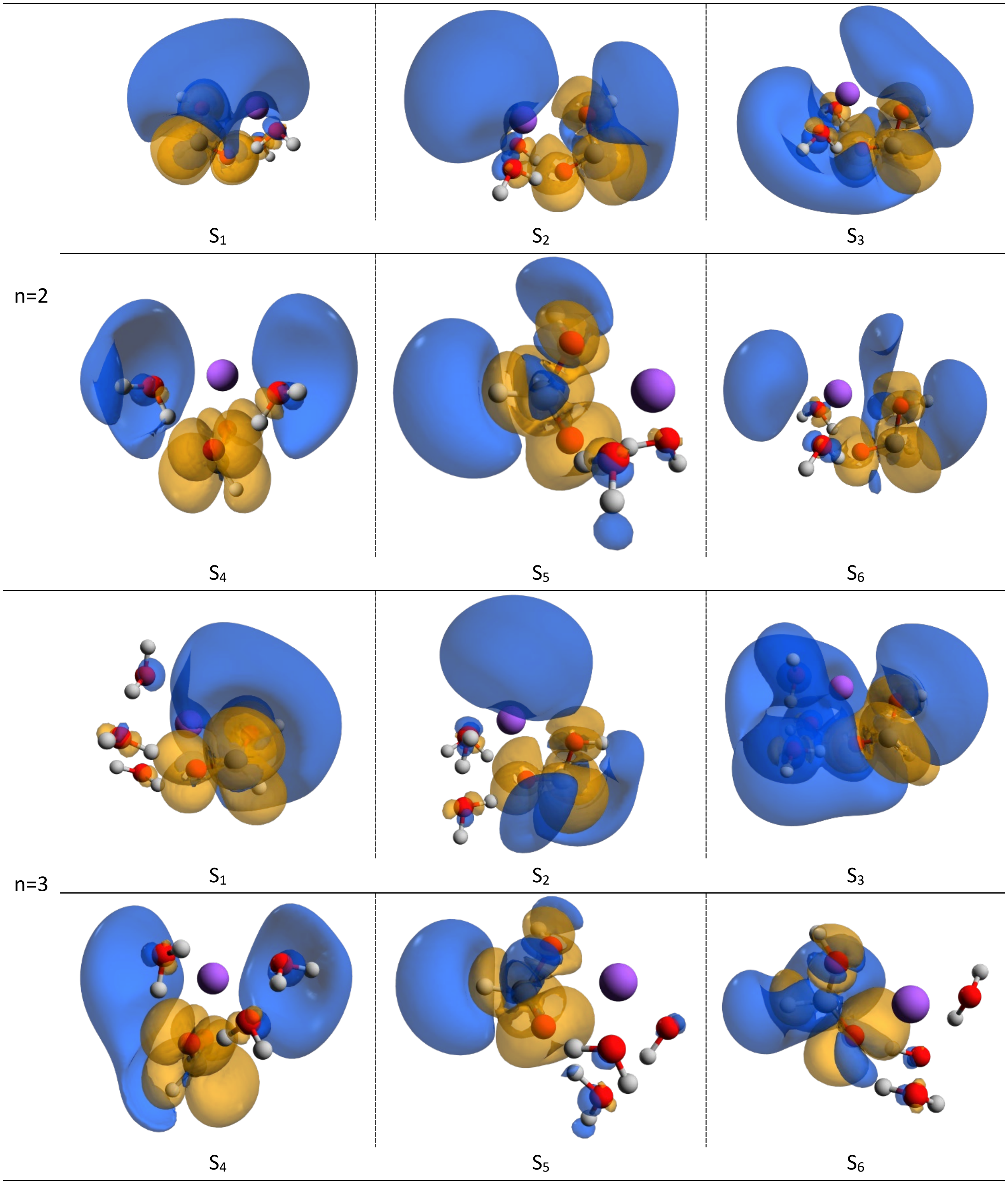
n=2
S1
S2
S3
S4
S5
S6
n=3
S1
S2
S3
S4
S5
S6

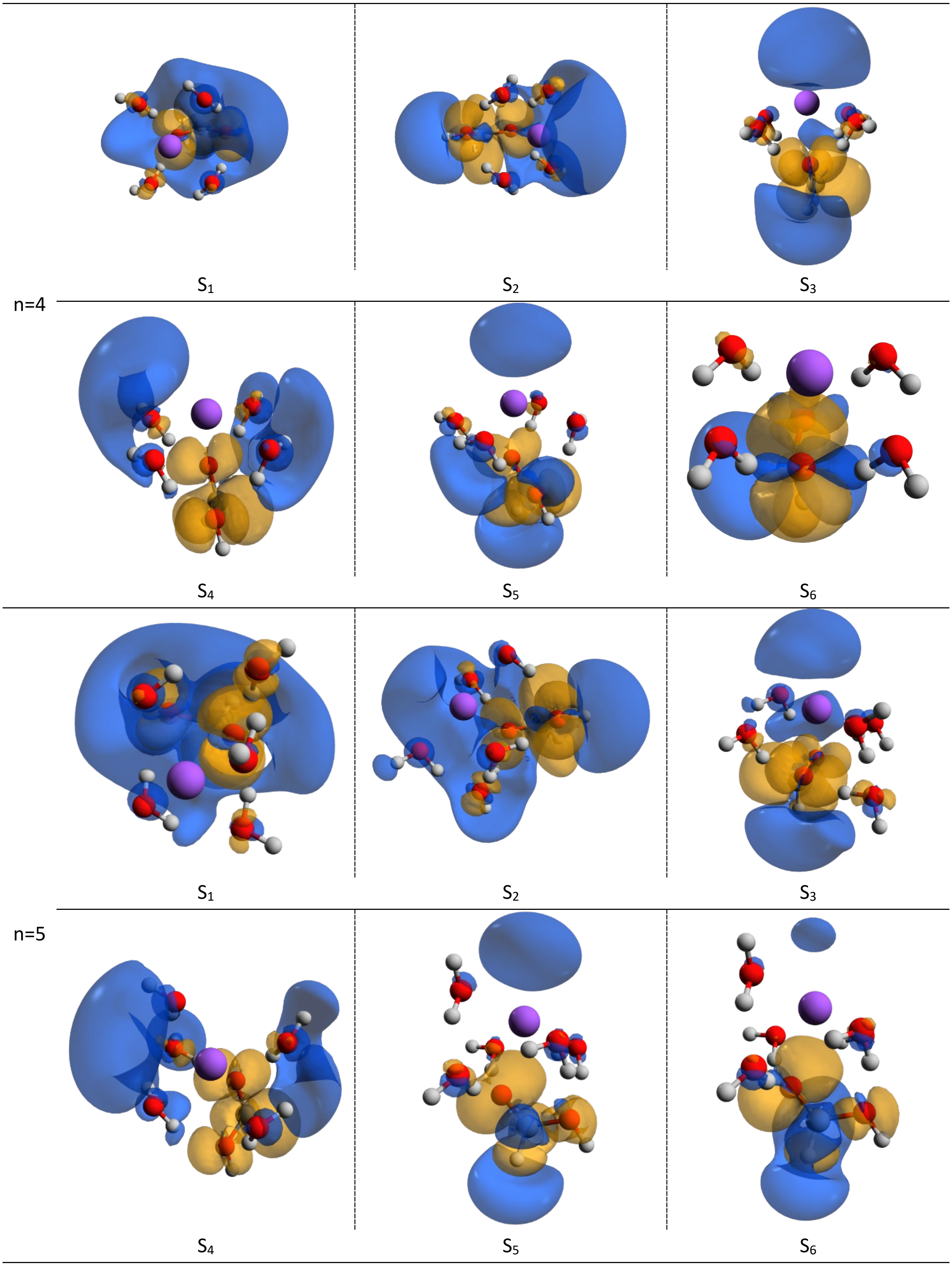
S1
S2
S3
n=4
S4
S5
S6
S1
S2
S3
n=5
S4
S5
S6

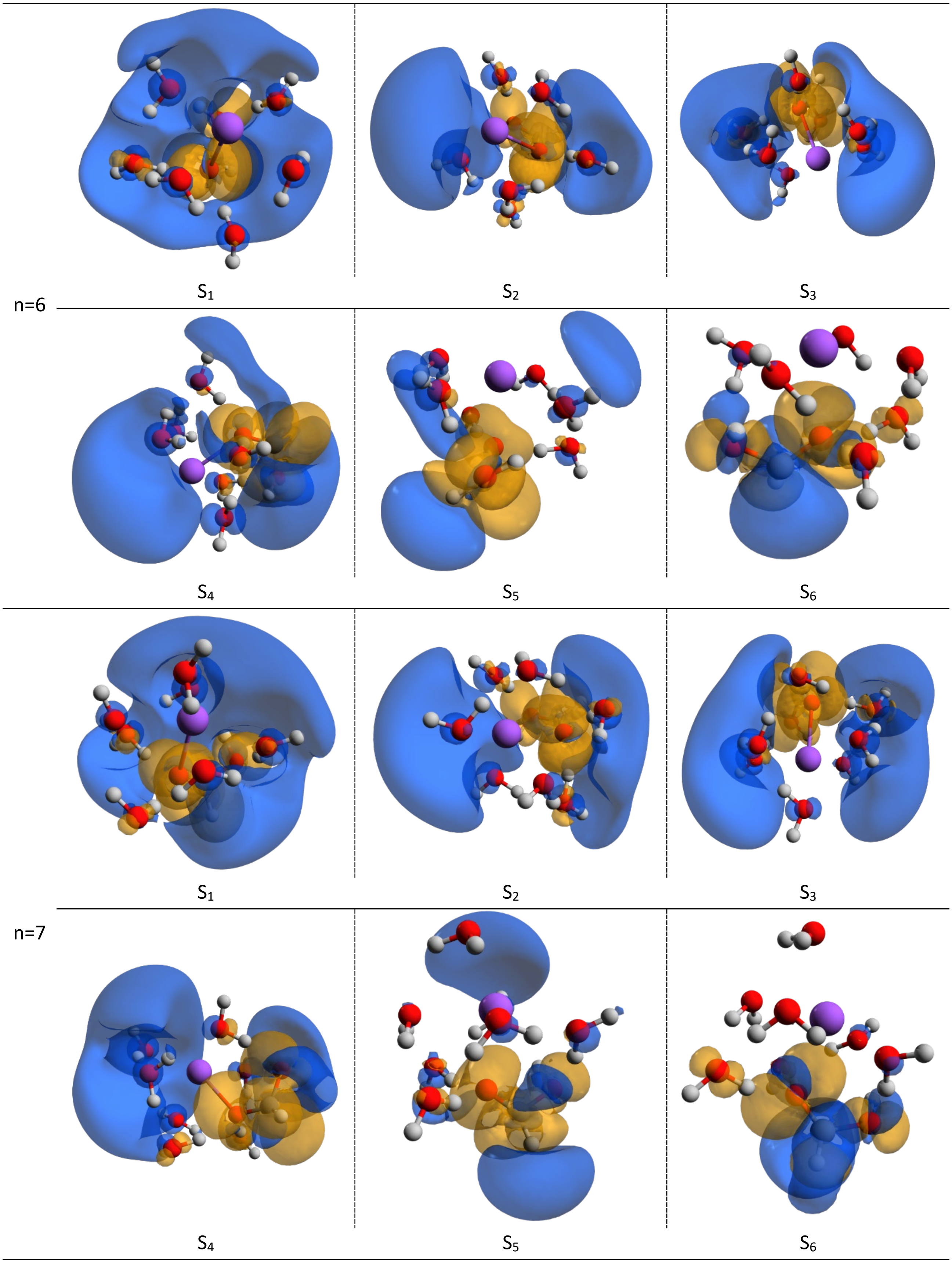
n=6
S1
S2
S3
S4
S5
S6
n=7
S1
S2
S3
S4
S5
S6

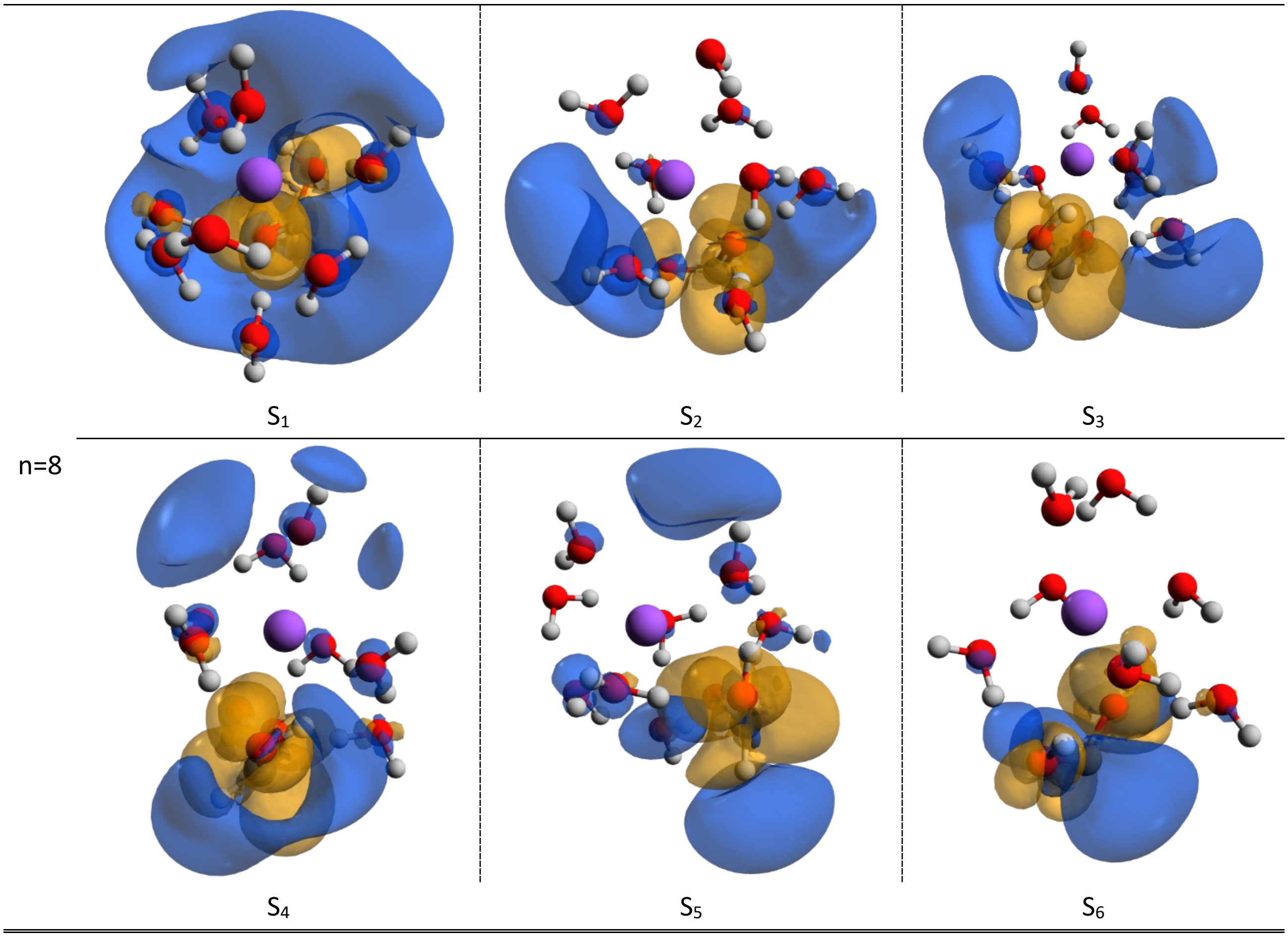


**Figure S4**. The natural difference orbital (NDO) profiles (orange=hole, blue=electron) compiled for the first six electronic excited states of Na···FA···$(H_2O)_n$ (n = 0,8) mixed clusters obtained at ωB2PLYP/def2-TZVPD level of theory.

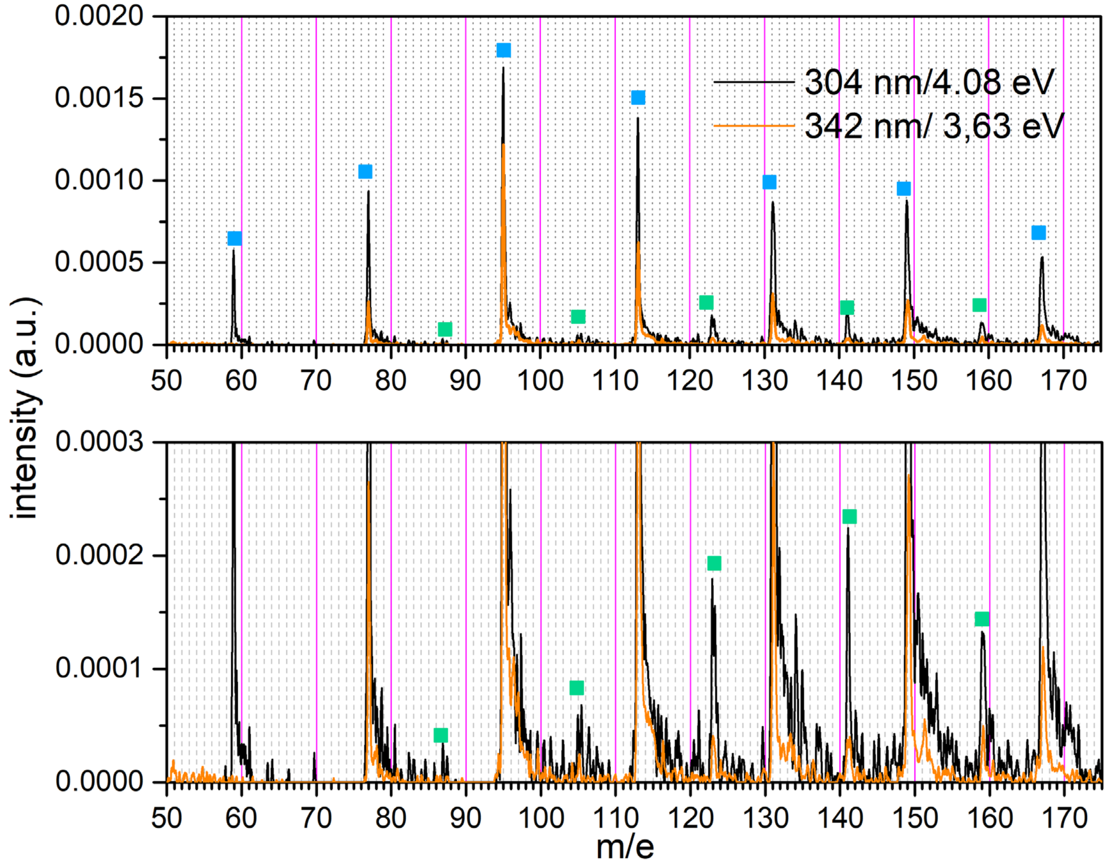


**Figure S5 a)** Mass spectrum expanded from 50 to 175 Da

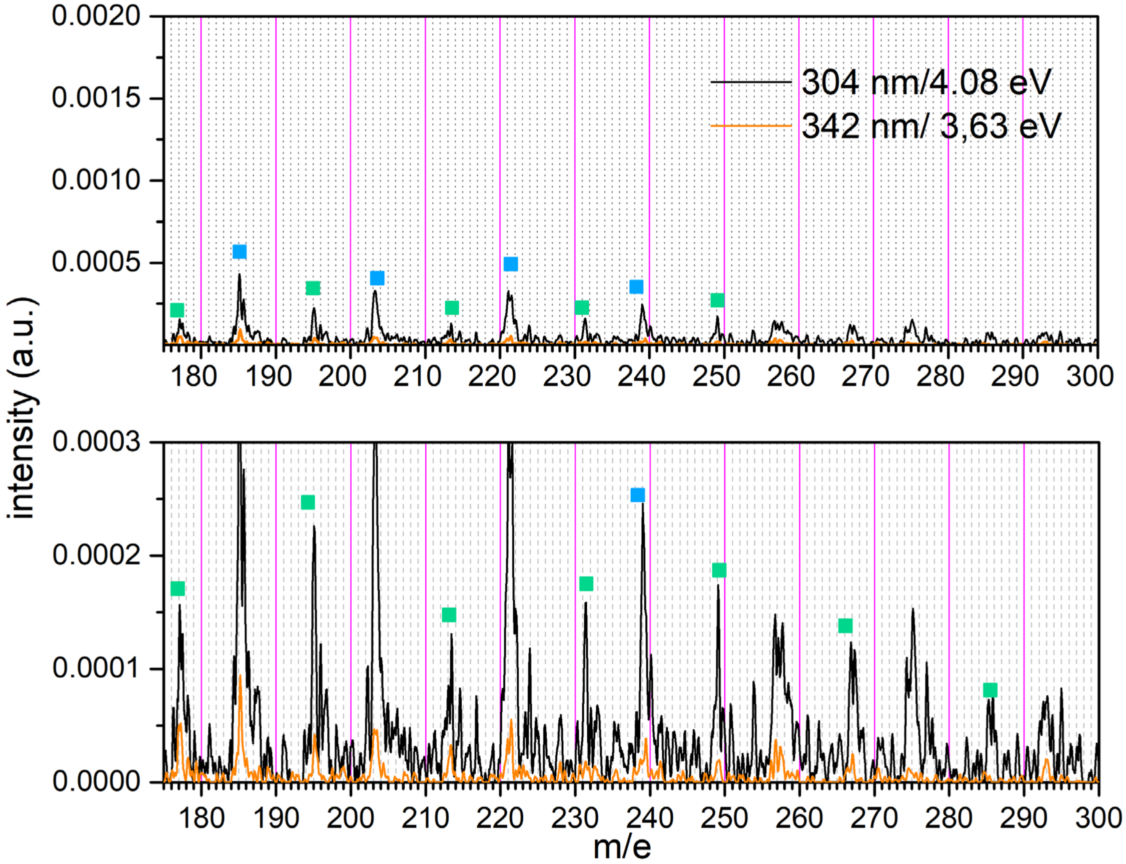


**Figure S5 b)** mass spectrum expanded from 175 to 300 Da
